# Beneficial Effects and Toxicity Studies of Xian-ling-gu-bao on Bone Metabolism in Ovariectomized Rats

**DOI:** 10.3389/fphar.2017.00273

**Published:** 2017-05-22

**Authors:** Hao Wu, Qingxiang Zhong, Jing Wang, Man Wang, Fang Fang, Zhi Xia, Rongling Zhong, Houcai Huang, Zhongcheng Ke, Yingjie Wei, Liang Feng, Ziqi Shi, E. Sun, Jie Song, Xiaobin Jia

**Affiliations:** ^1^Affiliated Hospital of Integrated Traditional Chinese and Western Medicine, Nanjing University of Chinese Medicine Nanjing, China; ^2^Key Laboratory of New Drug Delivery System of Chinese Materia Medica, Jiangsu Province Academy of Chinese Medicine Nanjing, China; ^3^College of Pharmacy, Anhui University of Chinese Medicine Hefei, China; ^4^College of Nursing, Huanghai University Qingdao, China; ^5^Laboratory Animal Center, Jiangsu Province Academy of Chinese Medicine Nanjing, China

**Keywords:** XLGB, ovariectomized rats, osteoporosis, toxicity test, OPG/RANKL

## Abstract

Xian-ling-gu-bao (XLGB) is a well-known patented traditional Chinese prescription widely used to treat osteoporosis, osteoarthritis, aseptic bone necrosis, or climacteric syndrome. However, recent reports have suggested that XLGB may cause liver injury in humans. In the present study, we aimed to evaluate the efficacy of XLGB in the prevention of osteoporosis in the zebrafish and ovariectomized (OVX) rats, both of which have been used as osteoporosis models. The safety of XLGB after long-term administration to OVX rats was also assessed. OVX rats were administered by oral gavage 270 mg/kg (recommended daily dose), 1350 mg/kg, and 1800 mg/kg of XLGB for 26 weeks. Bone mineral density, relative bone surface to bone volume, relative bone volume to total volume, trabecular number, mean trabecular thickness, and mean trabecular spacing in OVX rats were examined at the end of the 26-week dosing period. Additionally, OPG and RANKL expression in the femur were determined by western blot and immunohistochemical staining. To evaluate the safety of XLGB, body weight, hematology, serum biochemistry markers related to toxicology, and organ histopathology were determined in each group of OVX rats. Conversely, the zebrafish was treated with prednisolone to induce osteoporosis in the embryo. Disodium etidronate was used as a treatment control. XLGB was shown to be effective in preventing osteoporosis in both the OVX rats and the prednisolone-treated zebrafish. Similarly, XLGB increased OPG protein and decreased RANKL protein in OVX rats. Interestingly, no obvious toxicity was observed in the heart, liver, kidney, small intestine, or stomach at dosages of up to 1800 mg/kg after treating the OVX rats for 26 weeks. XLGB was shown to be very effective in treating osteoporosis in OVX rats. No obvious toxicity or adverse effects developed in OVX rats at dosages up to 1800 mg/kg, which is equivalent to six times the daily-recommended dose. Therefore, XLGB should be considered a good option for the treatment of post-menopausal osteoporosis.

## Introduction

Osteoporosis can significantly affect the quality of life of millions of senior citizens in our country. As the population grows, osteoporosis can have a major impact on the healthcare system. Osteoporosis is a chronic disease (Cornelius et al., 2014). Traditional Chinese Medicine (TCM) is known for being a low-cost option for chronic diseases and has fewer side effects than drug therapies (Li H. et al., 2016). Xian-ling-gu-bao (XLGB) has been used in TCM to treat osteoporosis. It consists of various ingredients, and it is administered as a capsule. The Chinese State Food and Drug Administration (SFDA) has officially approved it for use in TCM. It is orally administered in divided doses throughout the day. The ingredients of XLGB include Epimedii Herba, Dipsaci Asperoidis Radix, Psoraleae Fructus, Salviae Miltiorrhizae Radix, Rehmanniae Radix, and Anemarrhenae Rhizoma with Epimedii Herba as the emperor herb. Epimedii Herba, Psoraleae Fructus, and Anemarrhenae Rhizoma have all been shown to be effective in the prevention of osteoporosis (Kang et al., 2012; Jin et al., 2014; Wang et al., 2014).

Menopausal women have low estrogen levels and can develop osteoporosis. The ovariectomized rat model has been widely used to evaluate the effects of drugs in osteoporosis (Correa et al., 2016). Similarly, the zebrafish is an ideal vertebrate model for studying the toxicity of various drug therapies in tumor angiogenesis and bone formation (Zhang L.I. et al., 2016). The short generation time of the zebrafish offers advantages in developmental biology because of their transparent embryo and rapid organogenesis. To study treatment effects in osteoporosis the, zebrafish larva at 2–9 dpf (day post-fertilization) has been used.

Recent reports have suggested that the XLGB treatment may be associated with hepatotoxicity and have raised questions about its safety. The no-observed-adverse-effect level (NOAEL) for XLGB in normal Sprague-Dawley (SD) rats has been reported to be 1000 mg/kg. However, in OVX rats the NOAEL has not been studied. In a previous study, Wang et al. (2015) identified the bioactive fraction, in the XLGB extract, responsible for its osteogenic effects, by separating it into three fractions (XLGB-A, B, C). The fractions were administrated to 4-month old ovariectomized (OVX) mice for 6 weeks to evaluate their efficacy in treating osteoporosis. The evaluation was performed through microCT, biomechanical testing, and biochemical markers. In the present study, we aimed to verify the efficacy of XLGB in preventing osteoporosis by using the two osteoporosis models, the zebrafish and OVX rats. The safety of XLGB after long-term administration to OVX rats was also studied. The molecular mechanisms involved in the treatment of osteoporosis with XLGB were also explored in the OVX rat model by western blotting and immunohistochemical staining. The effects of XLGB in the liver were also studied.

## Materials and Methods

### Sample Preparation

The XLGB powder was supplied by Guizhou Tongjitang Pharmaceutical, Co., Ltd. The powder (20 g) was dissolved in 1000 mL of deionized water and subjected three times to ultrasonic extraction for half an hour. The mixed decoction was concentrated to 20 μg/mL for zebrafish. The XLGB powder for rat administration was suspended in deionized water.

### Animals and Experimental Groups

Fifty specific pathogen-free Sprague-Dawley (SD) rats were approved by the Animal Experimentation Ethics Committee of Jiangsu Provincial Academy of Chinese Medicine. All animals were purchased from the center of Nantong University and were housed four per cage at 24–27°C with a relative humidity of 55–60%. The room was maintained on a 12-h light/dark cycle with free access to water and lab chow. One week after the rats arrived, 10 female rats underwent bilateral laparotomy (sham), and 40 underwent bilateral ovariectomy (OVX; Zhang Z. et al., 2016). Four weeks after the procedure, OVX rats were randomly divided into the following four groups; OVX rats treated with low (270 mg/kg/d; XLGB-L, *n* = 8), medium (1350 mg/kg/d; XLGB-M; *n* = 8), and high (1800 mg/kg/d; XLGB-H, *n* = 8) XLGB dose. The remaining group of OVX rats was used as control (*n* = 8). At the end of the experiment, blood was collected and centrifuged at 3000 rpm for 10 min. The supernatant was collected and stored at -80°C until biochemical analyses were performed (Huang et al., 2013). The tissues from the femur samples were removed by gauze and stored with 4% neutral formaldehyde solution for micro-CT scanning before the immunohistochemical and western blot analyses (Anbinder et al., 2016). The organs were obtained after perfusing with normal saline (NS) and fixed in 4% paraformaldehyde for histological examination (Yan and Ye, 2015).

### Sample Preparation and Chromatography

The XLGB powder (0.2 g) was mixed with 8 mL of 60% methanol, and the mixture was subjected to ultrasonic extraction three times for 30 min and centrifuged at 12000 rpm for 5 min. The supernatant (50 μL) was injected into the UPLC system (Li Z. et al., 2016).

The measurements were performed using a Waters Acquity UPLC Iclass system (Waters, Milford, MA, United States) with a binary solvent delivery system, an autosampler, and a photodiode-array detection (PDA) system. The separation was carried out on a Waters ACQUITY BEH C_18_ column (2.1 mm × 100 mm I.D., 1.7 μm, Waters, Milford, MA, United States). The mobile phase consisted of (A) 0.1% formic acid in water and (B) acetonitrile. The UPLC elution conditions were as follows: 2% B (0–1 min), 2–20% B (1–10 min), 20–24% B (10–15 min), 24% B (15–24 min), 24–30% B (24–25 min), 30–80% B (25–40 min), 80–100% B (40–41 min), 100% B (41–42 min). The flow rate was maintained constant at 0.3 ml/min, and the temperature of the column was kept at 25°C (Geng et al., 2014). The compounds for chromatography were purchased from YuanYe Bio-Technology, Co., Ltd (Shanghai, People’s Republic of China). Other reagents were purchased from Nanjing Chemical Reagent, Co., Ltd (Nanjing, People’s Republic of China). All the reagents were of analytical grade.

### Monitoring

Clinical symptoms and mortalities were observed twice daily throughout the experiment. Body weight was measured weekly.

### Histomorphometric Analysis by Micro-CT

The left femur was analyzed by a high-performance *in vivo* micro-CT Skyscan (Skyscan1176, Skyscan). The images of pixel size 35 μm were filed to subsequently perform qualitative and quantitative analysis of the bone. The three-dimensional (3D) images were rebuilt by the software (CTVol, Skyscan). There were 50 consecutive slices (1.5 mm) selected as the region of interest beginning 6.22 mm away from the distal femur growth plate to analyze the trabecular bone (Yamada et al., 2016). For the quantitative analysis, the following measurements were obtained by the software: bone volume/tissue volume (BV/TV), bone surface/bone volume (BS/BV), trabecular thickness (Tb.Th), trabecular number (Tb.N), mean trabecular spacing (Tb.Sp), and bone mineral density (BMD).

### Immunohistochemical Staining

To evaluate the changes of bone after treatment with XLGB, immunohistochemical analyses were performed to determine the protein expression of OPG and RANKL. The left distal femur was embedded in paraffin and cut into about 5 μm thick sections. The tissues were deparaffinized and rehydrated. The slices were subsequently washed with PBS three times and prepared in a complex phosphoesterasum for 15 min at 37°C. They were subsequently incubated overnight at 4°C with primary antibodies (dilution 1:200; Boster Corporation, Wuhan, China). Following the incubation, the slices were washed three times with PBS and incubated again with secondary antibodies for 30 min. The slices were treated for color development with 3, 3′-diaminobenzidine (Wu et al., 2016). All the sections were semi-quantitatively analyzed by Image-Pro Plus (IPP) version 6.0 software. The integrated optical density (IOD) was measured by the staining in eight fields in each section on the images at 400× magnification, the average IOD as scored by three blind examiners was used for statistical analysis.

### Western Blot Analysis

Tissues from the right femur were lysed with RIPA lysis buffer containing protease and phosphatase inhibitors. The samples were incubated on ice for 30 min and separated by SDS-polyacrylamide gel electrophoresis (10%). The samples were then transferred onto polyvinylidene fluoride membranes and blocked in 5% milk for 1 h. Immunoblotting was subsequently performed by incubating the membranes overnight at 4°C with a primary antibody raised either against RANKL (1:1000), OPG (1:1000), or GAPDH (1:1000). The membranes were washed three times with TBST and incubated with the secondary antibody (1:5000) in the presence of horseradish peroxidase (Song et al., 2016). The experiments were repeated three times with different samples. The densities of the bands were determined using image analysis software Gel-pro Analyzer 4.5.

### Zebrafish Maintenance and Embryo Production

Wild-type zebrafish (*Danio rerio*) were maintained in groups of 30 (female: male = 1:1) in 10 L tanks at 27°C with continuous water exchange under 14-h light/10 h dark cycles. The fish were fed once or twice daily with parasites or flake food. On the day before collecting the *Danio rerio*, a Petri dish was placed at the corner of the tank with a sieve above to separate the *Danio rerio* from the adult zebrafish for a night. The Petri dish was taken out and rinsed with deionized water for several times until there were no feces or impurities, and incubated (Pérez-Schuster et al., 2016).

### Zebrafish Experimental Design

As previously reported, 25 μM of prednisolone exposure is an effective method for inducing osteoporosis in zebrafish. In our study, doses of 10, 1, and 0.1 ng/mL of XLGB were used to study its effects in preventing osteoporosis. Larvae at 3 days post-fertilization (dpf) were moved into 24-well plates (Corning) containing embryo medium (5 mM NaCl, 0.17 mM KCl, 0.33 mM CaCl_2_, 0.33 mM MgSO_4_, 5% methylthionine chloride) with each dose of XLGB and 25 μM prednisolone (Feng et al., 2015). The final volume of the solution was about 1000 μL in each well with eight zebrafishes. Disodium etidronate (15 mg/L) was used as a treatment control. All the solutions were replaced with fresh solutions through 9 pdf. The zebrafish larvae were anesthetized with MS-222 and fixed in 4% PFA overnight, after washing with 0.5% KOH/10% glycerol, the fish larvae were stained with 0.02% Alizarin stain/0.5% KOH/10% glycerol and shaken for 45 min. Following the staining period, a solution of 0.5% KOH/50% glycerol was used to distain. The larvae were subsequently bleached with 0.5% KOH/3% H_2_O_2_ for an appropriate time. Pictures of the processed larvae were obtained by using a fluorescence stereomicroscope (Olympus, Tokyo, Japan).

### Blood Analysis

Blood analyses were conducted at the end of the experiment. The animals were fasted but had access to water for 12 h prior to collecting the blood sample. The blood was collected from the rat’s orbit before they were anesthetized. The blood samples were collected into either heparinized centrifuge tubes or dried non-heparinized tubes. The non-heparinized blood was allowed to coagulate before being centrifuged and the serum separated. Aspartate aminotransferase (AST), alanine aminotransferase (ALT), alkaline phosphatase (ALP), total bilirubin (TBIL), blood urea nitrogen (BUN), and creatinine (CREA) were measured by a Biochemical Automatic Analyzer Hitachi 7080 (Hitachi, Ltd, Tokyo, Japan).

Hematologic values, obtained from the heparinized blood, included red blood cell (RBC), white blood cell (WBC), reticulocytes (RET), neutrophils (NE), lymphocytes (LYM), monocytes (MON), eosinophils (EOS), basophils (BAS), hemoglobin concentration (HGB), hematocrit (HCT), mean corpuscular volume (MCV), mean corpuscular hemoglobin (MCH), mean corpuscular hemoglobin concentration (MCHC), platelet count (PLT), red cell distribution width (RDW), platelet distribution width (PDW), mean platelet volume (MPV), and the plateletcrit (PCT).

### Hematoxylin-Eosin Staining

The safety of XLGB was evaluated by hematoxylin-eosin (HE) staining. Tissue samples from the heart, liver, kidney, small intestine, and stomach were fixed with 10% formalin in NS for 24 h before they were embedded in paraffin. After that, sections of about 4 μm thickness were made by using a microtome and stained with hematoxylin-eosin according to the manufacturer’s instructions (Song et al., 2015). The histopathologic changes in the staining images were observed using a light microscope (BX43, Olympus, Japan) with 100× magnification.

### Statistical Analysis

Data were expressed as mean ± standard deviation unless indicated otherwise. Differences between multiple groups were analyzed by ANOVA with the Bonferroni *post hoc* test. Differences between two groups were analyzed by the unpaired Student’s *t*-test. *p*-Value of < 0.05 was considered statistically significant.

## Results

### Ultra High-Performance Liquid Chromatography Analysis

**FIGURE 1:**
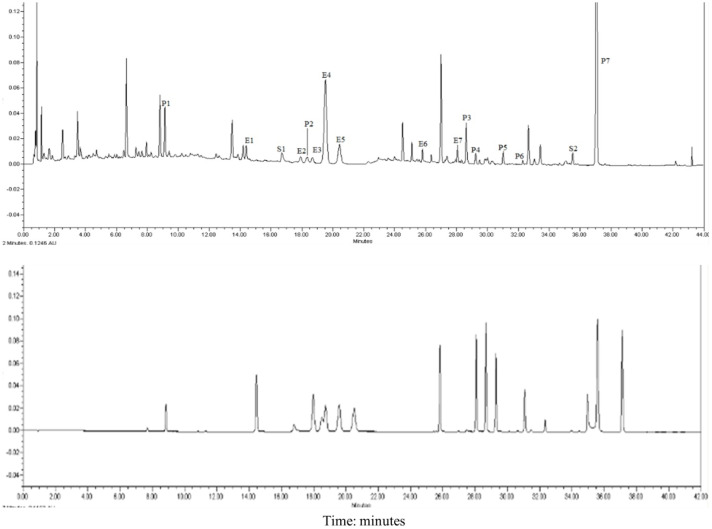
**UPLC chromatograms of XLGB.** E1 Epimedoside A; E2 Epimedin A; E3 Epimedin B; E4 Epimedin C; E5 Epimedoside; E6 Baohuoside II; E7 Baohuoside I; P1 Psoralen glycoside; P2 Psoralen; P3 Neobavaisoflavone; P4 Bavachin; P5 Psoralidin; P6 Isobavachalcone; P7 Bakuchiol; S1 Salvianolic acid B; S2 Tanshinone IIA.

Seven compounds were from Epimedii Herba (E1–E7), seven were from Fructus psoralea (P1–P7), and two were from Salvia miltiorrhiza (S1–S2). As shown in **Figure 1**. Most of the ingredients from XLGB are flavonoids, which are the most commonly used components for the treatment of osteoporosis.

### Clinical Observations

**FIGURE 2:**
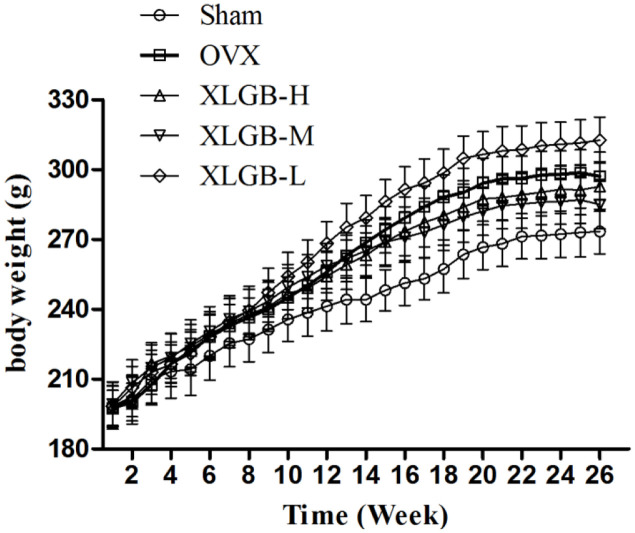
**The effects of oral administration of XLGB for 26 weeks on mean body weights, body weight was expressed as mean ± SD**.

There were no deaths observed between the treatment and control groups. No side effects were observed with dosages up to 1800 mg/kg XLGB. Similarly, mean body weight (**Figure 2**) was not significantly different between groups.

### Radiologic and Histologic Evaluations

**FIGURE 3:**
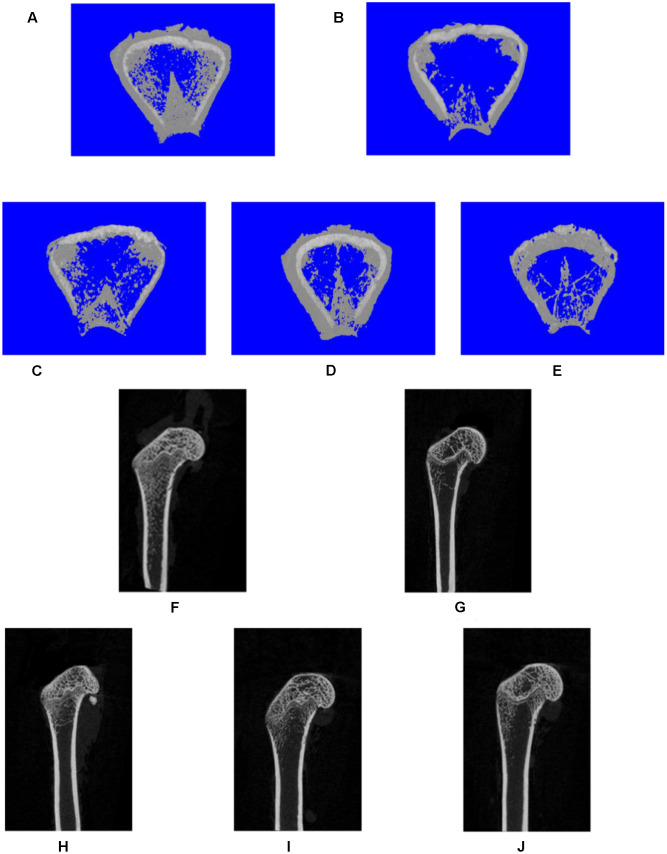
**Representative three-dimensional** **(A–E)** or two-dimensional **(F,J)** micro-CT images of trabecular bone from the distal femur with the sham and different OVX treatments. Three-dimensional: **(A)** sham group; **(B)** OVX group; **(C)** XLGB-H (2.57 g/kg/d); **(D)** XLGB-M (1.93 g/kg/d); **(E)** XLGB-L (0.39 g/kg/d). Two-dimensional: **(F)** sham group; **(G)** OVX group; **(H)** XLGB-H (2.57 g/kg/d); **(I)** XLGB-M (1.93 g/kg/d); **(J)** XLGB-L (0.39 g/kg/d).

**FIGURE 4:**
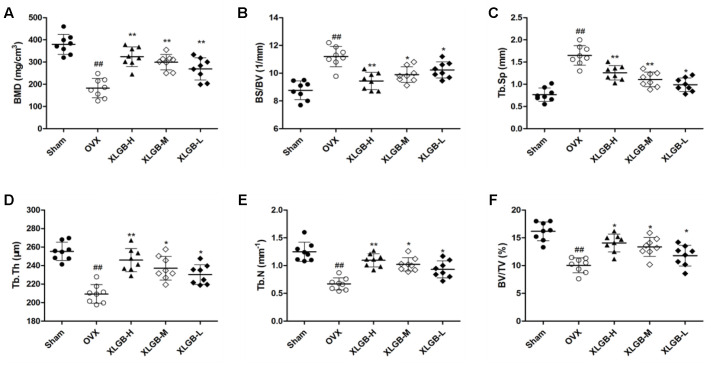
**Micro-CT analysis of (A)** BMD (bone mineral density), **(B)** BS/BV (bone surface/bone volume), **(C)** Tb.Sp (mean trabecular spacing), **(D)** Tb.Th (trabecular thickness), **(E)** Tb.N (trabecular number), **(F)** BV/TV (bone volume/tissue volume). Bone characteristics of sham (*n* = 8), OVX (*n* = 8), XLGB-H (*n* = 8), XLGB-M (*n* = 8), and XLGB-L (*n* = 8) SD rats after treatment for 26 weeks. Each value is expressed as mean ± SD. ^##^*p* < 0.01 compared with sham group by Student’s *t*-test; ^∗^*p* < 0.05, ^∗∗^*p* < 0.01 compared with OVX group by Student’s *t*-test.

To assess the mass and microarchitecture of the bone, the three-dimensional image of the distal femur with trabecular and two-dimensional images of the longitudinal plane were carried out using micro-CT (**Figure 3**). The parameters including BMD, BV/TV, Tb.N, Tb.Sp, BS/BV, and Tb.Th of each group are shown in **Figure 4**. The results showed that, following the ovariectomy, all the parameters for the OVX group were significantly different when compared to control (*p* < 0.01), except BS/BV. This shows that the ovariectomy was successful in creating the osteoporosis model for our studies. After 26 weeks of treatment, the BMD, BV/TV, Tb.N, and Tb.Th measurements of the XLGB treated groups were significantly higher than those of OVX mice (*p* < 0.05). Conversely, the BS/BV measurements of the XLGB group were significantly lower than those of OVX mice (*p* < 0.05). However, no differences in response to the incremental doses of XLGB that were administered were observed. These results suggest that XLGB has a positive effect on bone health in OVX mice. However, the effect was not dose-dependent.

### Effect of XLGB on the Zebrafish Osteoporosis Model

**FIGURE 5:**
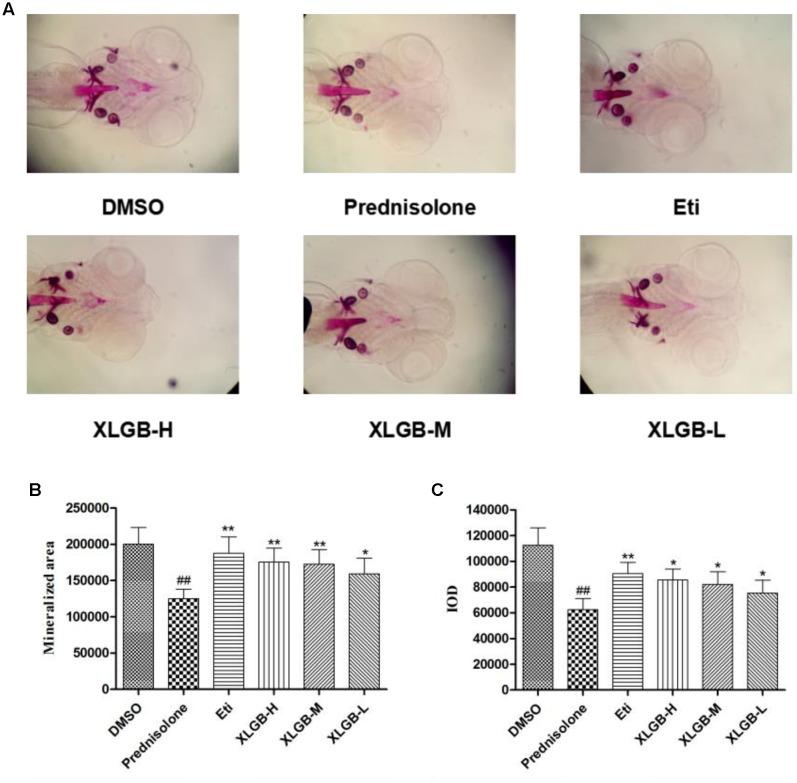
**Effect of different concentrations of XLGB groups of zebrafish larvae at 9 days post-fertilization (dpf) by ventral view of alizarin red whole-mount preparations **(A)**.** Effect of different concentrations of XLGB groups on mineralized area **(B)** and integrated optical density (IOD) **(C)** of mean pixel number of zebrafish larvae from 4 to 9 dpf stained with alizarin red. *n* = 6 or more, mean ± SD. ^##^*p* < 0.01 compared with sham group by Student’s *t*-test; ^∗^*p* < 0.05, ^∗∗^*p* < 0.01 compared with OVX group by Student’s *t*-test.

To determine the effect of XLGB on bone formation, we examined the development of osteoblasts exposed to prednisolone using alizarin red staining. The pictures showed that the mineralization area in the XLGB groups were significantly larger than that of the prednisolone treated group (**Figure 5A**). To validate the effect of incremental doses of XLGB in preventing osteoporosis, we compared the mineralized area (**Figure 5B**) and integrated the optical value for the density (**Figure 5C**) as measured by Image J software. The results showed that the mineralized area and IOD of the treated groups was significantly smaller than that of the DMSO group. This shows that prednisolone effectively induced osteoporosis in the zebrafish. With 6 days of XLGB treatment, the mineralized area and IOD area were significantly increased compared to the control group (*p* < 0.05), and the effect was dose-dependent.

### Immunohistochemical Staining

**FIGURE 6:**
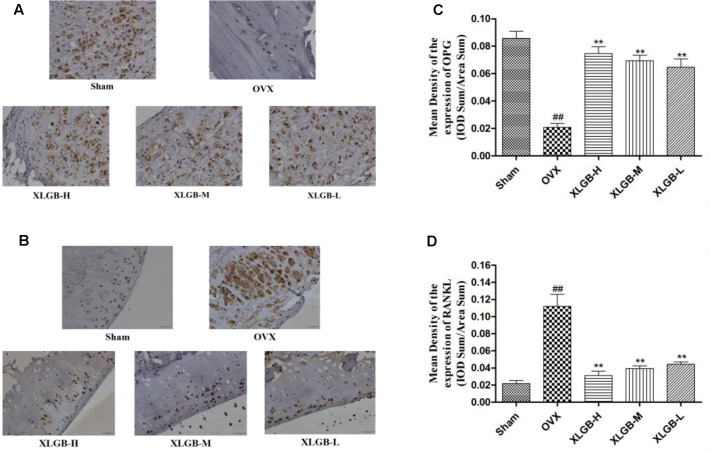
**Representative immunohistochemical staining of the femora from osteoporotic rats.** **(A)** OPG protein expression. **(B)** RANKL protein expression. **(C)** Relative expression of OPG protein levels. **(D)** Relative expression of RANKL protein levels. ^##^*p* < 0.01 compared with sham; ^∗∗^*p* < 0.01 compared with OVX.

The immunohistochemical staining assay was used to assess the OPG and RANKL protein expression of the femur in each group. As shown in **Figure 6**, OPG was significantly reduced while the RANKL protein expression was increased markedly in the OVX group compared to the sham group. These results show that osteoporosis developed in response to the ovariectomy. However, the development of osteoporosis was significantly (*p* < 0.01) inhibited by XLGB throughout the experiment.

### Western Blot

**FIGURE 7:**
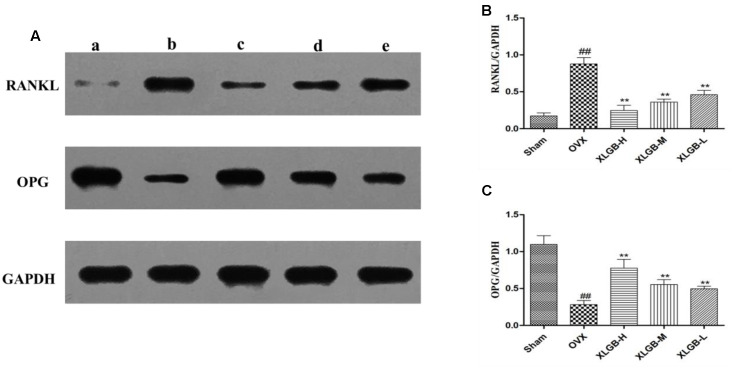
**Western blot analysis of the femora from osteoporotic rats.** **(A)** A sham group; b OVX group; c XLGB-H (2.57 g/kg/d); d XLGB-M (1.93 g/kg/d); e XLGB-L (0.39 g/kg/d). **(B)** RANKL protein level. **(C)** OPG protein level. Data are shown as the means ± SD, *n* = 3. ^##^*p* < 0.01 compared with sham; ^∗∗^*p* < 0.01 compared with OVX.

To measure the levels of protein of OPG and RNKL in the femur, a western blot was used. The results showed the same trend as that of the immunohistochemical staining (**Figure 7**). The expression of OPG was decreased while the expression of RANKL was increased in the OVX group compared with the sham group (*p* < 0.01). However, XLGB significantly (*p* < 0.01) inhibited these changes in protein expression compared to the OVX group. These observations suggest that XLGB plays an effective role in modulating the expression of OPG and the RANKL protein. In summary, the results showed that XLGB may regulate the expression of OPG and RANKL proteins in response to the ovariectomy.

### Hematologic Parameters

The hematopoietic system can be vulnerable to medications and serves as an important index of overall well-being for both humans and other animals. The results of the XLGB treatment on the hematologic parameters of the experimental and the control rats are presented in **Table 1**. Based on the hematologic analyses, there were no obvious differences between the control and the XLGB group. The treatment with XLGB did not cause a significant change in any of the biochemical markers compared to those in the control group.

**Table 1:** Hematological values in rats orally treated with XLGB.

		Hematological values				
	
Parameter	Unit	Sham	OVX	XLGB-H	XLGB-M	XLGB-L
RBC	10^9^/L	8.21 ± 1.92	7.32 ± 1.62	6.45 ± 1.81	6.93 ± 1.91	7.31 ± 2.11
WBC	10^9^/L	4.11 ± 1.35	3.27 ± 1.30	3.01 ± 1.28	3.62 ± 1.39	3.77 ± 1.67
RET	%	4.12 ± 1.12	3.49 ± 1.08	3.32 ± 1.16	3.83 ± 1.31	4.02 ± 1.19
NE	%	3.1 ± 1.01	3.42 ± 0.83	3.84 ± 1.42	3.67 ± 1.43	3.65 ± 1.24
LYM	%	77.12 ± 17.01	80.16 ± 17.14	82.34 ± 17.65	83.64 ± 18.01	80.12 ± 15.21
MON	%	4.68 ± 1.32	4.14 ± 1.12	4.88 ± 1.09	4.33 ± 1.25	5.01 ± 1.32
EOS	%	1.54 ± 0.36	1.72 ± 0.41	1.82 ± 0.47	1.85 ± 0.49	1.89 ± 0.56
BAS	%	2.17 ± 0.42	2.36 ± 0.57	2.43 ± 0.39	2.71 ± 0.48	2.62 ± 0.51
HGB	g/L	142.11 ± 25.32	159.32 ± 22.32	161.13 ± 23.75	166.12 ± 26.11	166.12 ± 27.09
HCT	%	35.88 ± 6.74	37.81 ± 6.12	39.14 ± 5.36	36.19 ± 6.21	40.12 ± 7.03
MCV	fL	49.21 ± 11.82	51.14 ± 13.23	52.44 ± 14.86	57.96 ± 12.01	60.23 ± 14.01
MCH	Pg	22.45 ± 5.26	21.73 ± 6.12	24.36 ± 6.54	26.32 ± 6.99	25.12 ± 5.69
MCHC	g/L	358.36 ± 52.39	342.17 ± 49.32	326.81 ± 60.69	365.22 ± 53.10	311.22 ± 56.19
PLT	10^9^/L	1012 ± 257.82	936.12 ± 246.12	1036.39 ± 230.15	1123.01 ± 267.32	1022.91 ± 281.32
DW	%	17.21 ± 4.11	14.25 ± 5.12	15.36 ± 5.15	16.37 ± 4.98	18.27 ± 5.02
PDW	%	28.25 ± 3.68	30.26 ± 4.12	33.24 ± 5.26	36.22 ± 7.69	35.74 ± 8.36
MPV	fL	7.21 ± 0.35	8.11 ± 0.42	9.87 ± 0.56	10.42 ± 0.85	12.57 ± 3.21
PCT	s	1.11 ± 0.20	0.82 ± 0.12	1.24 ± 0.36	1.41 ± 0.39	1.57 ± 0.65


*Red blood cell count (RBC), white blood cell count (WBC), reticulocytes (RET), neutrophils (NE), lymphocytes (LYM), monocytes (MON), eosinophis (EOS), basophils (BAS), hemoglobin concentration (HGB), hematocrit (HCT), mean corpuscular volume (MCV), mean corpuscular hemoglobin (MCH), mean corpuscular hemoglobin concentration (MCHC), platelet count (PLT), red cell distribution width (RDW), platelet distribution width (PDW), mean platelet volume (MPV), the plateletcrit (PCT).*

### Serum Characteristics

**FIGURE 8:**
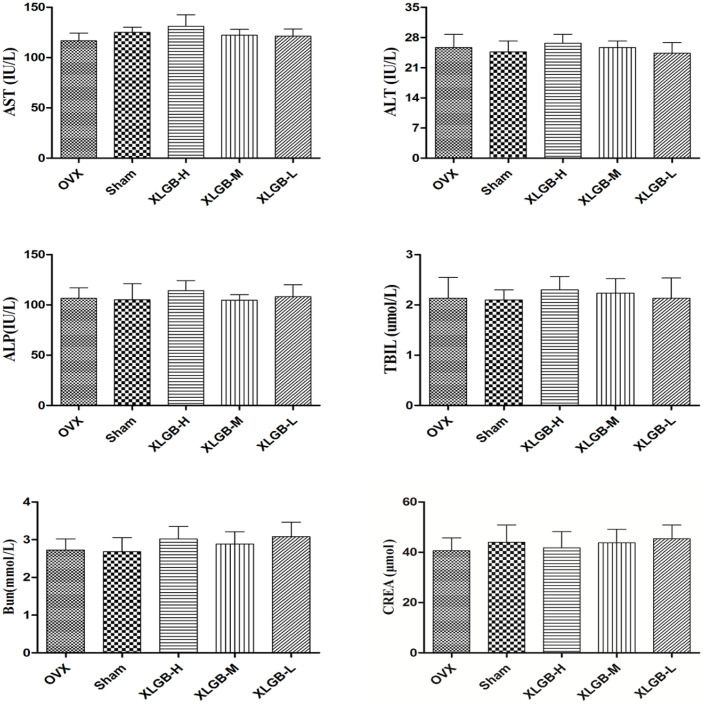
**Serum biochemical parameters of OVX rats treated with XLGB.** Data were expressed as means ± SD (*n* = 5 or more).

The serum biochemistry profile on liver enzymes is shown in **Figure 8**. There was no significant change in serum liver enzyme levels between the XLGB-treated group and the OVX group. This suggests that the long-term use of XLGB for the prevention of osteoporosis does not affect liver or kidney function.

### Toxicity Studies

**FIGURE 9:**
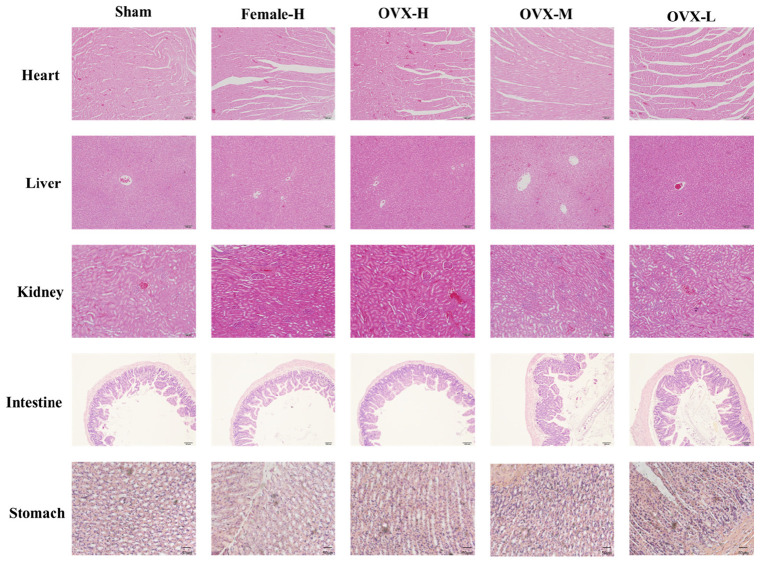
**The sections of main organ were obtained and stained with HE (magnification 200×)**.

To evaluate the side effects and toxicity of XLGB, we studied the histopathologic characteristics of tissues from organs including heart, liver, kidney, small intestine, and stomach. As shown in **Figure 9**, XLGB caused small pathologic changes in some of the tissues. For example, the histopathology revealed slight steatosis in the liver. However, similar changes were observed in the sham and the OVX group. In addition, a slight inflammation of the kidneys was observed in each experimental group. To study the effects of XLGB in the gastrointestinal system, we evaluated the histopathologic changes of the small intestine and stomach. The results showed that there were no obvious changes between the sham, OVX and the treatment group. Therefore, XLGB, when administered, at doses up to 1800 mg/kg does not cause toxicity.

## Discussion

The world population is getting older, and with that, we can expect to see an increase in chronic diseases. One such disease is osteoporosis. The number of osteoporosis cases in the world is growing rapidly and continues to have an impact on the healthcare system (Anbinder et al., 2016). Since osteoporosis is a chronic disease, TCM can have certain advantages because patients experience fewer side effects and, thus, it is suitable for long-term use (Feize et al., 2016). XLGB is one of the TCM formulas, which has been used in the treatment of osteoporosis.

In the present study, two types of animal models, which included the zebrafish and OVX rats, were used to evaluate the efficacy of XLGB. The osteoporosis model that is induced by bilateral ovariectomy in rats is similar to that of post-menopause in humans during which they experience high bone loss. As expected, the results showed that OVX rats had lower bone density than those in the sham group. However, the degree of osteoporosis was significantly lower in the group that was administered XLGB for 26 weeks. The analyses performed on the femur of rats showed that OVX rats treated with XLGB had higher BMD, BV/TV, Tb.Th, Tb.N, Tb.Sp, and lower BS/BV than the OVX group.

Bone mineral density, which is an important marker for bone strength, serves as a basis to predict the risk of fracture (Zhao et al., 2016). The trabecular bone is the extension of the cortex, and it is used as the golden standard to diagnose osteoporosis. Conversely, the cancellous bone is where hematopoiesis takes place (Chen et al., 2016). Therefore, the trabecular number, thickness, and spacing are important parameters in judging the quality of the bone. In the present study, the XLGB treatment significantly improved the trabecular number and thickness and decreased its spacing. These observations suggest that when osteoporosis is induced in rats, XLGB can prevent the changes caused by osteoporosis in the trabecular bone.

Bone volume to total volume represents the bone volume per tissue volume, which is also used as a gold standard to determine the number of bones. Similarly, Tb.Th, Tb.N, and Tb.Sp correspond respectively to thickness, number, and trabecular spacing. It is well-known that the number and thickness of the trabecular bone are positively correlated with the quality of the bone. Conversely, the trabecular spacing is negatively correlated. In this study, the OVX group had increased spacing, decreased thickness, and trabecular bone number, which is consistent with bone deterioration. However, when XLGB was administered for half a year, it prevented those changes. In our preliminary study (data not shown), XLGB at 90, 180, 270 mg/kg/d showed that the improvement in osteoporosis symptoms was dose-dependent.

Another aim of the study was to examine the safety of XLGB when administered long-term. Thus, a higher range of dosages, which included 270, 1350, and 1800 mg/kg/day were administered. The 270 mg/kg/d dose represented the daily-recommended dose. However, the degree of toxicity was not dose-dependent. We speculate that the 270 mg/kg dose was optimal. It is possible that the saturation of the target tissue, by competitive binding, could have contributed to this phenomenon.

Osteoporosis is a complex biochemical process, which involves a balance between the osteoclast-mediated bone resorption, and osteoblast-mediated bone formation. A variety of physiological and pathological signals will influence the function of osteoclast and osteoblast, which can then affect the structure and function of the bone. Bone remodeling requires a precise balance between bone formation and resorption (Sato et al., 2016). It is well-known that RANKL is a cytokine which can induce the differentiation of progenitor cells into mature osteoclast (Jiang et al., 2016). Conversely, OPG is an osteoprotegerin which can block the binding of a RANK ligand (RANKL) to the RANK receptor (Kurinami et al., 2016). The discovery of the RANKL/RANK/OPG pathway has opened new treatment avenues for osteoporosis treatment in recent years (Holloway et al., 2002). The ratio of OPG/RANKL is key to maintaining a balance between bone formation and resorption (Yuan et al., 2017). In the OVX group, the ratio of OPG/RANKL decreased significantly. This means that the speed of resorption became faster than its formation, which results in bone metabolism imbalance, and can decrease BMD and subsequently cause osteoporosis. A variety of cytokines regulates the balance between osteoclasts and osteoblasts (Abdelgawad et al., 2016). Therefore, it is necessary to study the pharmacological effects of bone metabolism at the molecular level. In our study, immunohistochemical staining and western blotting were used to detect the expression of OPG and RANKL in femur tissue. The results showed that the ratio of OPG/RANKL decreased in the OVX group. However, the ratio improved with the administration of XLGB. The results of the present study showed that XLGB was associated with an increase in the OPG/RANKL ratio, which may inhibit bone loss and suggests a possible mechanism for XLGB action.

In the present study, no obvious hepatotoxicity was found in the rats after 6 months of XLGB administration in both healthy and treated rats, as demonstrated by serum biochemistry assays and HE staining. However, in light of species differences, preclinical animal toxicity studies may not accurately predict human toxicity. Due to the low occurrence rate of side effects observed with XLGB, we hypothesize that idiosyncratic hepatotoxicity, drug-drug interactions, and gene polymorphism of cytochrome P450 may be involved. However, further studies are warranted. Unfortunately, to date, no animal model is well-accepted to be used to study idiosyncratic hepatotoxicity. This is a big obstacle when studying XLGB safety.

## Ethics Statement

All animal experiments were reviewed and approved by the Animal Care and Use Committee of the Animal Laboratory Center of Jiangsu Research Institute of TCM and adhered to the guidelines of the Guide for the Care and Use of Laboratory Animals.

## Author Contributions

HW, QZ, JW, and MW performed the research. JS and XJ designed the research study. FF, RZ, ZX, and HH analyzed the data. HW wrote the paper. All authors approved the final version of the manuscript.

## Conflict of Interest Statement

The authors declare that the research was conducted in the absence of any commercial or financial relationships that could be construed as a potential conflict of interest.
