# Corrigendum: Beneficial Effects and Toxicity Studies of Xian-ling-gu-bao on Bone Metabolism in Ovariectomized Rats

**DOI:** 10.3389/fphar.2020.570876

**Published:** 2020-11-09

**Authors:** Hao Wu, Qingxiang Zhong, Jing Wang, Man Wang, Fang Fang, Zhi Xia, Rongling Zhong, Houcai Huang, Zhongcheng Ke, Yingjie Wei, Liang Feng, Ziqi Shi, E. Sun, Jie Song, Xiaobin Jia

**Affiliations:** ^1^ Affiliated Hospital of Integrated Traditional Chinese and Western Medicine, Nanjing University of Chinese Medicine, Nanjing, China; ^2^ Key Laboratory of New Drug Delivery System of Chinese Materia Medica, Jiangsu Province Academy of Chinese Medicine, Nanjing, China; ^3^ College of Pharmacy, Anhui University of Chinese Medicine, Hefei, China; ^4^ College of Nursing, Huanghai University, Qingdao, China; ^5^ Laboratory Animal Center, Jiangsu Province Academy of Chinese Medicine, Nanjing, China

**Keywords:** XLBG, ovariectomized rats, osteoporosis, toxicity test, OPG/RANKL

In the original article, there was an error in **Figure 9**. An image of the high-dose group was inserted into the middle-dose group in the stomach row. The correct **Figure 9** appears below.

**Figure 9:**
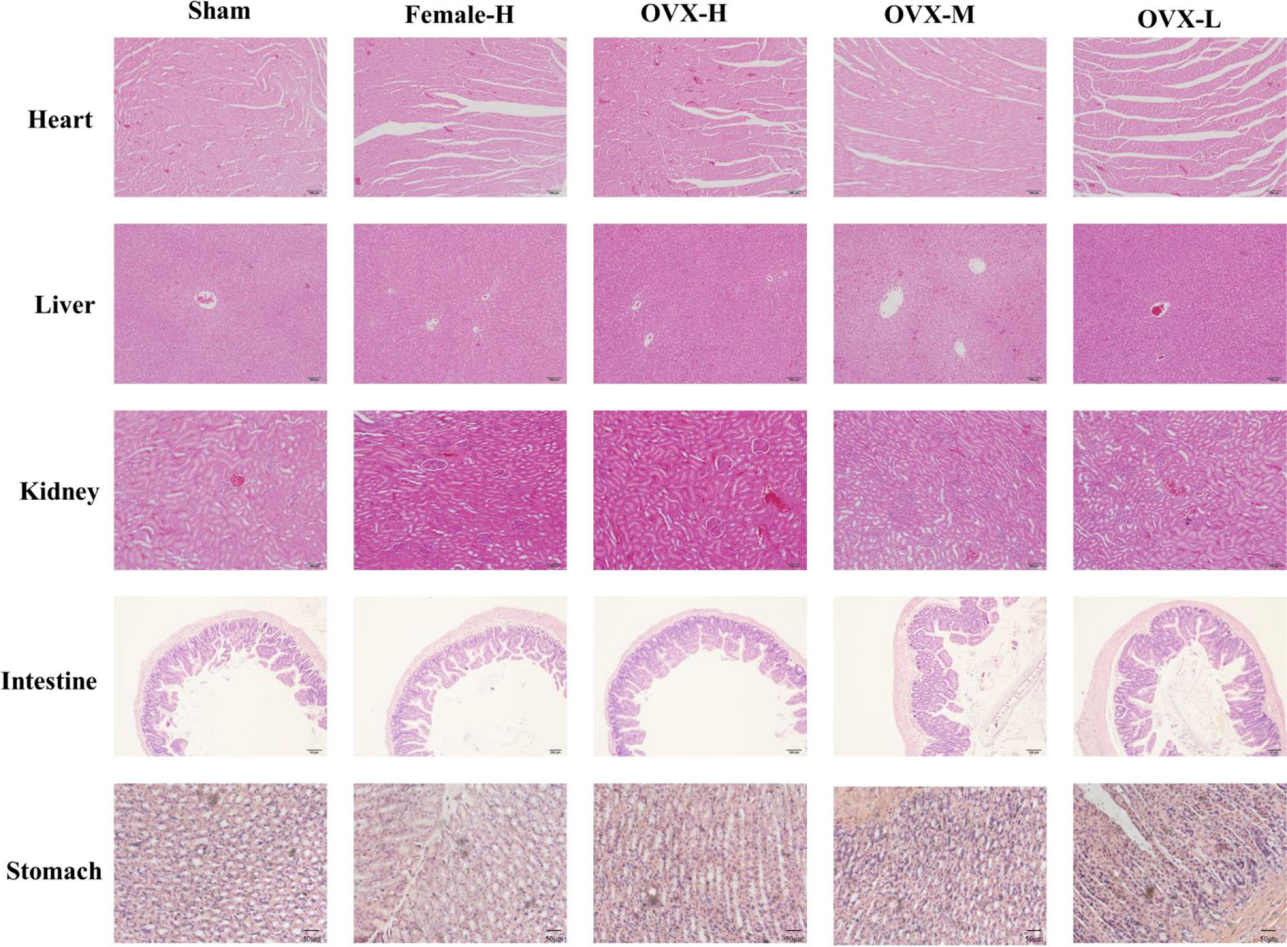
The sections of main organ were obtained and stained with HE (magnification 200×).

The authors apologize for this error and state that this does not change the scientific conclusions of the article in any way. The original article has been updated.

